# Supplementary material for: Comprehensive Evaluation of Lipid Nanoparticles and Polyplex Nanomicelles for Muscle-Targeted mRNA Delivery
Source: Pharmaceutics. 2023 Sep 7;15(9):2291. doi: 10.3390/pharmaceutics15092291 (PMC10536695; doi:10.3390/pharmaceutics15092291)
Supplement: Supplementary file 1 [file pharmaceutics-15-02291-s001.zip › pharmaceutics-2578150-supplementary.pdf]

Supplementary Materials

Comprehensive Evaluation of Lipid Nanoparticles and Polyplex Nanomicelles for Muscle-targeted mRNA Delivery

Xuan Du[1], Erica Yada[1,2], Yuki Terai[1], Takuya Takahashi[1], Hideyuki Nakanishi[1], Hiroki Tanaka[3], Hidetaka Akita[3] and Keiji Itaka[1,4,*]

[1]Department of Biofunction Research, Institute of Biomaterials and Bioengineering, Tokyo Medical and Dental University (TMDU), Tokyo 101-0062 , Japan;
[2]NANO MRNA, Co., Ltd. Tokyo 104-0031, Japan;
[3]Graduate School of Pharmaceutical Sciences, Tohoku University, Sendai 980-8578, Japan;
[4]Clinical Biotechnology Team, Center for Infectious Disease Education and Research (CiDER), Osaka University, Osaka 565-0871, Japan

Table S1. The particle size, polydispersity (PDI), and zeta-potential of LNP and nanomicelle used in this study.

	Particle size (nm)	PDI	ζ-potential (mV)
LNP	84.87	0.160	-3.47
nanomicelle	52.83*	0.162*	0.071*

* citation [12] in the main text

Figure S1

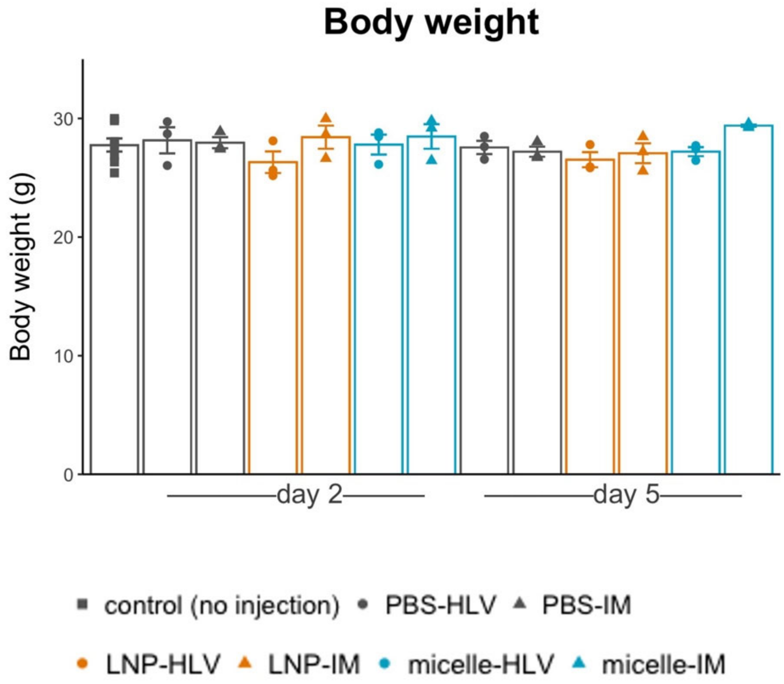

Figure S1. Body weight of the mice 2 and 5 days after administration (n = 3 and n = 6 for the control group). Data are presented as mean ± SEM.
